# When Light Is Crucial, but Wind Is Rather Trivial: A Basil Case Study

**DOI:** 10.3390/plants13223221

**Published:** 2024-11-16

**Authors:** Efterpi Florou, Angela Politi, Evangelia Andreadaki, Konstantinos Vrakas, Hariklia Spaliara, Alexandros Neli, Christina Eleni Koulopoulou, Athanasios Koulopoulos, Filippos Bantis, George Zervoudakis

**Affiliations:** 1 Department of Agriculture, University of Patras, 30200 Mesolongi, Greece; efiflorou99@gmail.com (E.F.); politi.aggela@gmail.com (A.P.); euoula8@gmail.com (E.A.); kostasvrakas99@gmail.com (K.V.); spaliarax@gmail.com (H.S.); alexandrosneli@hotmail.com (A.N.); tkoulop@upatras.gr (A.K.); fbantis@uowm.gr (F.B.); 2 Department of Chemistry, National and Kapodistrian University of Athens, 15784 Athens, Greece; xristinaak00@gmail.com

**Keywords:** anthocyanins, basil, carotenoids, chlorophyll, growth, light, pigments, wind

## Abstract

Light intensity and wind are critical environmental factors of abiotic stress on plants, triggering a considerable number of morphological and physiological responses. This study tested the hypothesis that different light and wind conditions (full sunlight ± wind, shade ± wind) would affect the leaf content of photosynthetic pigments and anthocyanins, as well as the plant height, the fresh and dry weight of the aboveground part, and Water-Use Efficiency on *Ocimum basilicum* plants. About 16 days after the application of the different conditions, all leaf pigments of the shaded plants exhibited increased contents compared to the lightened ones. Subsequently, this response was enhanced until the 39th day, which was the final day of the experiment. Furthermore, shaded plants grew taller, although their fresh and dry weight and Water-Use Efficiency were lower than that of the corresponding lightened ones. On the other hand, wind did not have any effect on pigment content. Concerning the plant growth indexes, reduced values were observed on the wind-treated plants. The above results demonstrate a considerable effect of light intensity while the applied wind seems to be mild enough to induce important plant responses, partially confirming the hypothesis studied.

## 1. Introduction

*Ocimum basilicum* L. (basil) is one of the most popular culinary herbs belonging to the Lamiaceae family. It is a native of Africa, India, and Asia, and it is cultivated in open fields, in temperate climates throughout the world. Basil is used in traditional medicines, and it is a popular herb in North American and Mediterranean diets. It is widely used in the food and cosmetic industries, as it contains phytochemical constituents with different pharmacological applications [1,2] such as essential oils [3,4] and antioxidant polyphenolic compounds with anticancer, antibacterial, antifungal, and anti-inflammatory capabilities [5,6].

Among the main environmental factors, solar radiation is the most significant one, regulating photosynthesis and influencing other physiological processes, and consequently, affecting the plant’s survival, growth, and adaptation. In any habitat the light intensity varies temporally (seasonally and diurnally) and spatially. Therefore, plants develop acclimation and plasticity to cope with the varying light regimes [7,8]. Most plant species can develop anatomical, morphological, physiological, and biochemical alterations in response to different light intensities [8,9,10]. These acclimation responses contribute to the optimization of acquisition and utilization of light. For example, the mesophyll of leaves exposed to high light intensities, is generally thicker due to an increased number of cell layers in palisade parenchyma compared to shade leaves [10]. On the other hand, plant leaves grown under low irradiance develop higher photosynthetic pigments contents, especially chlorophyll b, facilitating the absorption of diffused light in shaded environments and thus optimizing their light absorption efficiency [8,11,12,13,14]. A similar pattern has been observed for anthocyanins. Considering that anthocyanins are phenolic compounds with high antioxidant potential, there is a notable effect of anthocyanin–light interactions on high/low irradiance [14]. Concerning the plant biomass and the number of leaves, they seem to be lower under low light while plant height and leaf area are typically greater, compared to plants grown under more intense light conditions [9,12].

Wind is a ubiquitous but rather neglected environmental factor that has various effects on plants, affecting not only the development, architecture, and morphology of their aboveground part but also of their root system. Wind is known to improve the anchorage of plants by strengthening the development of roots, affecting the root system architecture and therefore the plant–soil interactions [15,16]. Terrestrial plants have adapted to survive under a range of wind patterns which cause changes in their chemical composition, physical structure, and morphology at all scales from the cell to the whole plant [17]. The impact of wind on plants depends on its speed, duration, and the extent to which wind can penetrate canopy layers. Sufficient wind speeds can affect both the plant’s growth, resulting in reductions in leaf and plant size (dwarfing) [18,19], and the plant’s physiological processes such as photosynthesis [15]. Moreover, wind alters heat and mass transfer, for example by increasing leaf transpiration rate through the reduction of boundary layer resistance. High winds can also damage the plant’s organs and tissues affecting the incidence of pests and diseases within crops [19]. On the other hand, a wind that is too weak or of short-duration pulses will not cause significant changes, especially on the aboveground part of the plant [16].

Under natural conditions, plants must simultaneously cope with multiple stresses, either abiotic or biotic ones. Therefore, it becomes increasingly interesting to investigate if and how plant responses caused by different stresses are interconnected. While the effect of light intensity has been studied on several plants [12,20], the impact of wind has not been thoroughly investigated. In this study, we investigated physiological and growth responses of basil, against different light intensity and wind conditions. In particular, the photosynthetic pigments and anthocyanin leaf content were investigated as well as the height and fresh and dry weight of plants grown under low and high light intensity, in both the presence and absence of wind. Considering that shade affects plants’ growth, morphology, and leaf pigmentation, usually provoking lower biomass but taller plants and a higher content of photosynthetic pigments, the aim of this research was to evaluate not only the effect of light intensity on basil but also its interaction with the wind effect. The bibliography is poor regarding the effect of wind on leaf pigmentation since it only been reported that chlorophyll and anthocyanin contents decreased by mechanical stimulus [21]. Thus, the aim of our research was also to evaluate the impact of wind on plant height and biomass, and to investigate its effect on leaf pigmentation.

## 2. Results and Discussion

**Figure 1:**
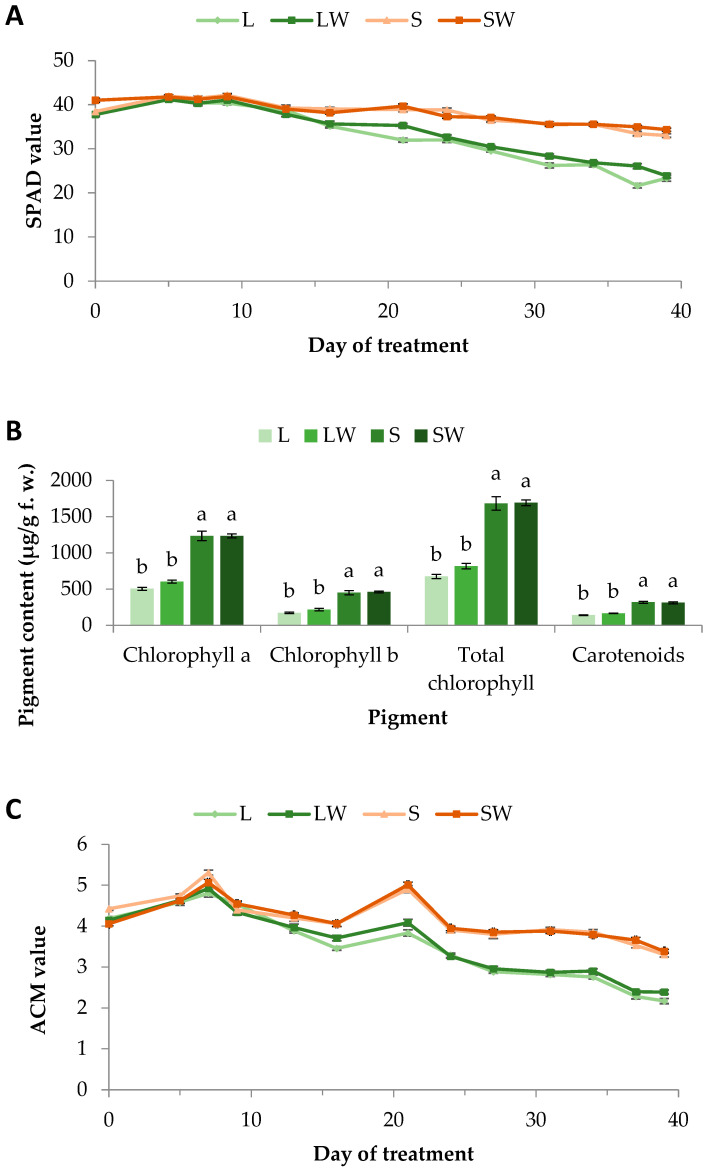
(**A**) Chlorophyll content measured by SPAD, (**B**) pigment concentration (chlorophylls and carotenoids), and (**C**) anthocyanin content measured by ACM of basil plants grown for 39 days under different light intensity and wind conditions. In (**B**), bars (±SE) followed by different letters are significantly different (*p* ≤ 0.05). In (**A**), standard errors ranged between 0.26 and 0.67, while in (**C**), standard errors ranged between 0.04 and 0.10.

All 28 plants (seven plants per treatment) grew normally and without physiological disorders. According to a 39-day period of SPAD measurements, the leaf chlorophyll content profiles of basil plants were similar for all four treatments until about D16. Thereafter, a large decrease in the chlorophyll content appeared in plants cultivated under full sunlight and irrespective of wind treatments, which lasted until the end of the experiment. Specifically, on D39 the decrease was 39 and 37% for L (Light treatment) and LW (Light + Wind treatment) plants, respectively, compared to the corresponding values of D0. Under shade, the chlorophyll content followed a similar pattern but at a lower rate, that is a 14 and 17% decrease to S (Shade treatment) and SW (Shade + Wind treatment) plants, respectively (Figure 1A). Therefore, at the end of the experiment, shaded plants exhibited 41–43% higher SPAD values than the lightened ones. The above results demonstrate that while the chlorophyll content decreases during basil aging, shading prevents the chlorophyll decrease.

The results above are in accordance with the photosynthetic pigment concentrations determined after acetone extraction. On D23, all pigments (chla, chlb, total chl, and carotenoids) in shaded plants were at least two-fold higher compared to the lightened ones (Figure 1B). Considering that the synthesis and/or degradation of chlorophyll occurs naturally with the presence of light, plants have adaptations to increase their light-use efficiency under different light conditions. Full sunlight can cause photooxidation of chlorophyll which can lead to chlorophyll degradation. On the contrary, under low light conditions (10–20% of full sunlight), plants optimize their light absorption efficiency by biosynthesizing larger amounts of photosynthetic pigments [8,13]. Moreover, in shaded plants the increase was larger for the chlb concentration than the chla one (Figure 1B). Specifically, chlb increased 164% and 129% in S and SW plants compared to L and LW ones, respectively, while the corresponding increases for chla were 144% and 105%. It has been reported that chlorophyll b is higher in shaded plants, as a result of extensive stacking of grana [22] facilitating the absorption of blue-violet and orange light from diffused light in shaded environments [14]. In accordance with our results, chlorophyll degradation under high light conditions has been mentioned by other researchers too. A gradual drop of leaf chlorophyll content in *Arabidopsis* was exhibited over time under high light conditions of 1000 μmol m^−2^ s^−1^ [23], while even only a 12 h exposure under strong light (100,000 lx) provoked chlorophyll degradation in strawberry leaves, compared to the corresponding chlorophyll content of plants exposed under 30,000 lx [24], proving the relationship between pigment content and light intensity. In addition, carotenoids participate as photoprotective agents in the light-harvesting complex of the photosystems which are damaged under the effect of high light intensity, leading to a reduction in the carotenoid content [25]. The same was observed in *Salvia officinalis* which exhibited 75% greater carotenoid content under the lowest sunlight intensity tested which was 25% of full ambient light [12]. Our experiment exhibits similar results since carotenoid content was about 80–120% higher in basil plants grown under 14% of full ambient light compared to the corresponding content in plants under full sunlight.

Anthocyanins are phenolic compounds with high antioxidant potential, and they are abundant in basil. Light properties such as intensity, quality, and photoperiod are known to considerably affect phenolic biosynthesis in plants including herbs and vegetables [26]. Although a positive effect of light on the anthocyanin content has been reported in other plants [27,28], in our case, anthocyanin content showed a similar trend to the SPAD measurements. Specifically, similar values were recorded for all treatments until about D16, while a significant decrease was observed thereafter for both light treatments compared to the shadow ones, irrespective of wind treatments. At the end of the experiment, shaded plants exhibited 41–52% higher anthocyanin content than the lightened ones. Additionally, on D39 the decrease was 48 and 42% for L and LW plants, respectively, compared to the corresponding values of D0. On the other hand, shade induced only 25 and 17% decrease to S and SW plants, respectively (Figure 1C). The great reduction under ambient light conditions can be attributed to anthocyanin’s high light photoprotective function which leads to the molecule’s gradual oxidation by reactive oxygen species [29]. It is known that under high light, reactive oxygen species such as H_2_O_2_ are produced in chloroplasts. H_2_O_2_ can spread out from the chloroplast membrane and subsequently enter the vacuole through the tonoplast. Peroxidases located in vacuoles can use anthocyanins as a substrate, oxidizing them and inducing the loss of their color [30]. The consensus has been that increased light results in increased anthocyanins and other flavonoids content. However, some authors have reported no change with different light treatments, while others had observed the opposite effect [31]. Besides that, temperature is also an environmental factor that plays an important role in regulating anthocyanin biosynthesis. High temperature reduces anthocyanin concentration, and transcripts of the genes of the anthocyanin biosynthetic pathway lead to colorlessness [32,33]. Our results are in contrast to the commonly referred anthocyanin accumulation under high light intensity conditions. Considering that our experiment was conducted in a greenhouse during May and June, the measured basil anthocyanin content is maybe the result of the combination effect of both high light and temperature conditions since the shaded plants were exposed to lower temperatures.

**Figure 2:**
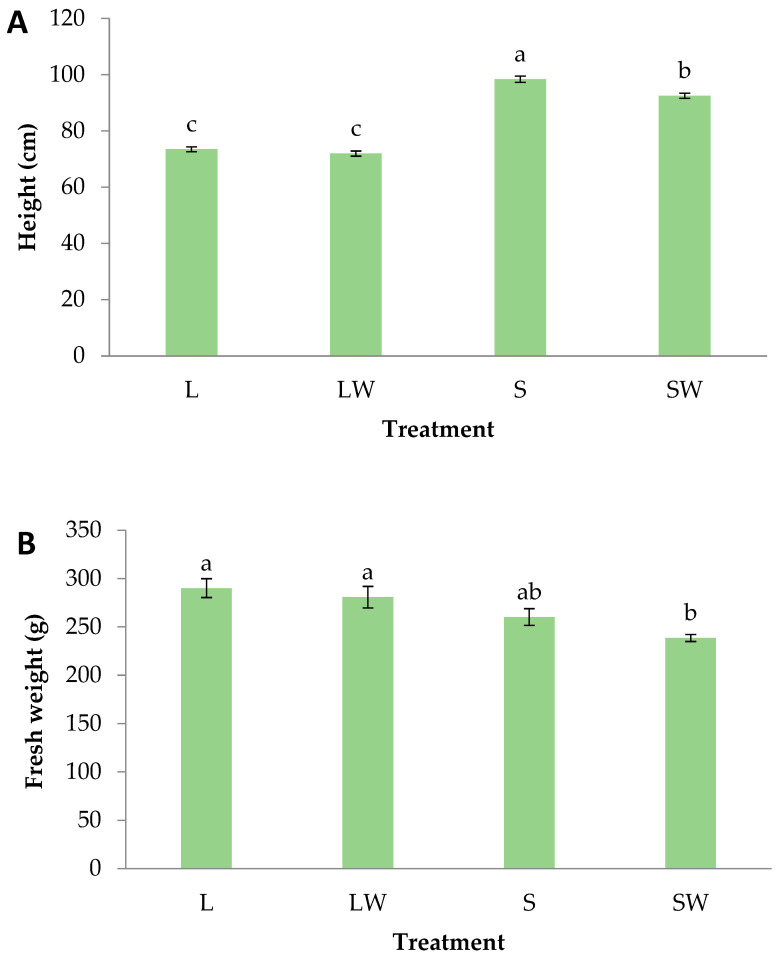

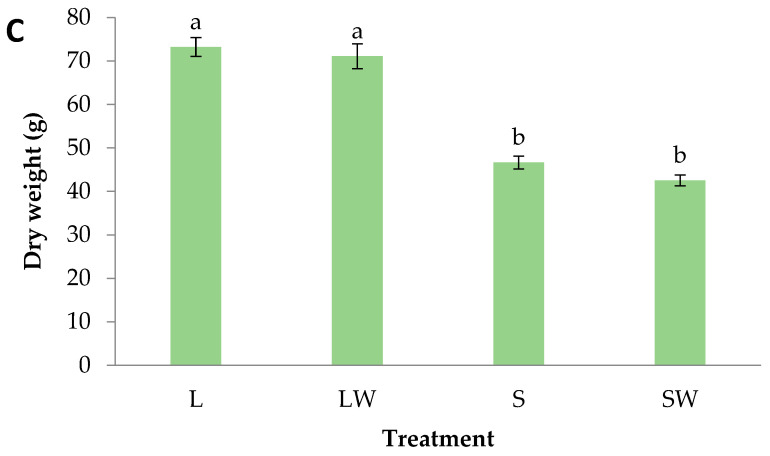
(**A**) Height, (**B**) fresh weight, and (**C**) dry weight of basil plants grown for 39 days under different light intensity and wind conditions. The treatments are L—Light, LW—Light and Wind, S—Shadow, SW—Shadow and Wind. Bars (±SE) followed by different letters are significantly different (*p* ≤ 0.05).

Stem height at the end of the experiment was significantly greater on shadowed plants. S plants were 34% higher than L ones and SW plants were 28% higher compared to LW ones (Figure 2A). The stem growth of S and SW individual plants implies a typical shade avoidance syndrome. Shade avoidance responses are modifications exhibited by plants grown under light intensity below the saturation level required by the specific species, when the abundance of the Phytochrome Interacting Factors (PIFs) promote the shade avoidance syndrome regulating the shade-induced gene expression [34,35]. On the other hand, under full light conditions, phytochromes promote the inhibition of PIFs, thus inducing transcriptional reprogramming, resulting in photomorphogenic development [36]. Typical modifications of shade avoidance syndrome include but are not limited to shoot elongation, leaf extension growth, and flowering initiation [37]. A similar response was reported for tomato plants since a decreasing height trend was exhibited when light intensity was increased from 50 to 300 μmol m^−2^ s^−1^ [38].

At the end of the experiment, the plants’ fresh weight seemed to be lower under shadowed conditions, which is 11% for S plants compared to L ones (not significantly different) and 15% for SW plants compared to LW ones (significantly different) (Figure 2B). On the other hand, dry weight showed a more acute response. Here, both shadowed treatments were significantly diminished compared to both lightened ones, with 37–41% lower values (Figure 2C). A similar observation was made for lettuce where no differences were found in fresh weight, but dry weight was significantly greater under ambient light compared to a shade cloth with 35% light transmission [39], while the maximum growth-promoting effect for lettuce is met at a light intensity of 400 μmol m^−2^ s^−1^ [40]. In our case, it is obvious that the shade cloth, which permits only 132 μmol m^−2^ s^−1^ transmission of ambient sunlight, limited the plants’ biomass accumulation since the light saturation point was not met. Similarly, tomato plants exhibited significantly less fresh and dry weight when grown under low light conditions [38].

**Figure 3:**
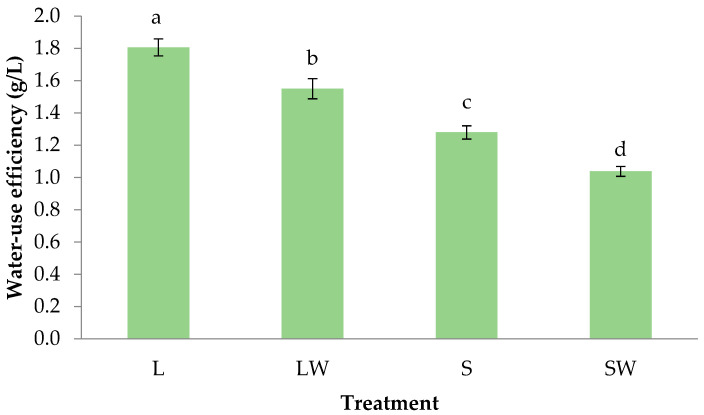
Water-Use Efficiency of basil plants grown for 39 days under different light intensity and wind conditions. The treatments are L—Light, LW—Light and Wind, S—Shadow, SW—Shadow and Wind. Bars (±SE) followed by different letters are significantly different (*p* ≤ 0.05).

Nowadays, water scarcity introduces the necessity to monitor and control the amounts of water needed by crops. WUE is an important parameter in vegetable production due to the high water requirements of summer crops. In our case, WUE was significantly different among all treatments with values showing the following trend: L > LW > S > SW (Figure 3). Specifically, L showed 18% greater WUE compared to LW, while S showed 27% greater WUE compared to SW. In addition, L and LW showed 40 and 50% greater WUE compared to S and SW, respectively. Our results are in accordance with a recent study which reported a gradual increase in WUE of lettuce under increasing light intensities ranging from 75 to 600 μmol m^−2^ s^−1^ [41]. Moreover, a study on *Sinapis alba* also showed that a high wind speed of 3.63–3.74 m s^−1^ leads to decreased WUE values in comparison to 0.31–0.37 m s^−1^ wind speed conditions [42]. Although our results are in accordance with the widespread perception that transpiration is increased under wind conditions, some authors have noted that transpiration may decrease in response to increasing wind speed under certain conditions [43]. Wind effects on plants occur both via mechanical effects and by influencing the turbulent transfer of heat, water vapor and CO_2_. Therefore, important interactive effects with different levels of CO_2_ and water supply are expected [42].

In general, wind treatments imposed minor effects on basil growth, irrespective of the light intensity. The SPAD and anthocyanin values were not significantly affected except for individual dates (Table 1). It is known that wind decreases plant height [44] but during our experiment only the stem height of SW was significantly lower (6%) compared to S, while the lightened plants were not affected. Moreover, even though there was no significant effect of wind on biomass accumulation, a tendency for reduced values was observed under the wind treatments. For example, L and S showed 3 and 9% greater fresh weight compared to LW and SW, respectively, while similar results are exhibited from the corresponding comparison of the dry weight values. A similar fresh weight trend was also reported in lettuce, where the most protected plants against the wind effect (close to the fence that surrounded the experimental system) exhibited enhanced growth compared to the most exposed ones [45]. Nevertheless, a slight wind is favorable for the limitation of pathogen infections. Indeed, in another study, nocturnal fanning with a wind speed of 0.4–1.5 m s^−1^ considerably suppressed the development of downy mildew in sweet basil [46]. There are not any previous findings in the literature about the effect of wind on the leaf pigmentation (chlorophylls, carotenoids, anthocyanins) of terrestrial plants, although it has been reported that mechanical stimulus (touch treatment) decreased the hypocotyl anthocyanin and chlorophyll content in *Carica papaya* [21]. On the other hand, there is little information about its effect on photosynthesis and transpiration. For example, it has been shown that gas exchange parameters are affected when wind speed is at least 6 m s^−1^ [47] while in our experiment the applied wind speed was only 0.98 m s^−1^, implying that the applied wind perhaps was mild enough to induce important plant responses.

## 3. Materials and Methods

### 3.1. Plant Material and Experimental Conditions

Twenty-eight young *Ocimum basilicum* L. (basil) seedlings of the same age were obtained from a local nursery at the end of April. They were transferred to an experimental greenhouse of the University of Patras in Amaliada (South-West Greece, 37°48′ N, 21°21′ E) and transplanted to 4 L pots filled with loamy sand soil. All plants were grown under the same environmental conditions during a two-weeks acclimation period; firstly for one week under a shade cloth (14% transmission of full ambient light) and subsequently for one week under greenhouse ambient sunlight.

The experiment was conducted from May 11th to June 19th and lasted 39 days (D39). Specifically, after two weeks of the acclimation period mentioned above, on D0 of the experiment, the plants were divided at random into four groups of seven plants per group, that is one group for each treatment. The first group remained under ambient sunlight (Light: L treatment), the second group remained under ambient sunlight and in the presence of wind generated by a household fan (Light + Wind: LW treatment), the third group was installed under a shade cloth permitting 14% transmission of ambient sunlight (Shade: S treatment), and the fourth group was installed under a similar shade cloth in the presence of wind generated by a household fan (Shade + Wind: SW treatment). The daily average Photosynthetically Active Radiation (PAR) under ambient sunlight or under the shade cloth was 942 μmol m^−2^ s^−1^ and 132 μmol m^−2^ s^−1^, respectively. These values are the corresponding averages of PAR measurements which were obtained every 10 min from dawn to dusk with two Quantum Sensors Model SQ-521 (Apogee Instruments, Logan, UT, USA).

Both plant groups of the wind treatments (LW and SW) were located semi circularly, and each plant was distanced 0.5 m from the corresponding fan. On the plants, the wind speed was 0.98 m s^−1^, measured with the FP111Β Global Water Flow Probe (Global Water Instrumentation, Gold River, CA, USA). The head of the fan was moving in an arc, stressing transiently the plants from the 1st to the 7th, and returning to the 1st again when a period was completed. Each period lasted 12 s.

The plants were irrigated daily in the afternoon. Each plant was irrigated until there was runoff from the pot. The average daily irrigation doses per plant during the 39 day experiment were 1050, 1210, 935, and 1080 mL for L, LW, S, and SW treatments, respectively. Fertilization was performed five times for all plants. The fertilization doses each time were 1 g of crystalline fertilizer [20–20–20 + 5] (N–P–K + Mg) per plant.

Upon completion of the experiment, 39 days after the treatments’ installation, the height was measured with a tape ruler from the plant base up to the apical bud. All the plants were harvested and the above ground part (shoots plus leaves) from each one was weighed. To obtain the dry weight, the samples were dried to constant weight in an oven at 70 °C for 72 h. The Water-Use Efficiency (WUE) of each plant was also calculated as follows: WUE = dry weight/total irrigation water.

### 3.2. Measurements of Photosynthetic Pigments and Anthocyanins

Nondestructive measurements of chlorophylls and anthocyanins were obtained with a SPAD 502DL chlorophyll meter (KONICA MINOLTA, Tokyo, Japan) and an ACM-200plus anthocyanin content meter (ADC BioScientific Ltd., Hoddesdon, UK), respectively. SPAD and ACM measurements are relative index values displayed by the corresponding meter, having a correlation to the pigment density. Leaf pigment measurements were conducted twice per week for all seven plants per treatment. The measurements were obtained on three randomly selected and completely expanded young leaves per plant, that is 21 leaves for each treatment.

In addition, leaf samples of the same physiological age as those used for nondestructive measurements were collected from each treatment. Each treatment sample consisted of seven leaves (one of each plant) wrapped in plastic bags and transferred immediately to the lab for photosynthetic pigment (chlorophyll a, chlorophyll b, carotenoids) content estimation. Two leaf discs (diameter 1.2 cm) were obtained from each leaf. The extraction procedure was carried out under dim light. The leaf discs were ground in porcelain mortar using 100% acetone and the resulting suspension was centrifuged (8000× *g*, 5 min, 4 °C). Chlorophyll and carotenoids contents were determined in acetone supernatants by using adjusted extinction coefficient and equations [48]. The absorbance was determined with a Shimadzu UV-1601 spectrophotometer (Shimadzu Corp., Kyoto, Japan). Four replicates of the above procedure were conducted for each experimental treatment during both D23 and D39 of the experiment.

### 3.3. Data Analysis

The results for all the measured variables were obtained from all seven plants per treatment (L, LW, S, SW) and were plotted as the mean ± standard error of mean (SE).

All data were plotted using Microsoft Office Excel and statistical analyses were carried out with GraphPad Prism v.9.0 (San Diego, CA, USA). Statistical differences were calculated using an ANOVA test with Tukey’s multiple comparison test. The statistical significance level was set at a = 0.05.

## 4. Conclusions

Shade affected the height, the biomass production, the WUE, and the pigment accumulation (chlorophylls, carotenoids, and anthocyanins). Specifically, the height was increased under the shade cloth, showing a typical shade avoidance response. Fresh and dry biomass, as well as WUE were lower under the shade cloth compared to the plants under the ambient light conditions. Chlorophylls, carotenoids, and anthocyanins showed a stronger decline under the ambient sunlight. On the other hand, wind did not affect the basil’s pigmentation but caused a decrease of WUE in both light treatments and of stem height under shade.

## Figures and Tables

**Table 1:** Comparison of the different treatments for each day of measurement (statistical significance according to one-way ANOVA).

		Day of Measurement												
	Treatment	0	5	7	9	13	16	21	24	27	31	34	37	39
SPAD	L vs. LW	ns	ns	ns	ns	ns	ns	**	ns	ns	ns	ns	***	ns
	L vs. S	ns	ns	ns	ns	ns	***	****	****	****	****	****	****	****
	L vs. SW	**	ns	ns	ns	ns	**	****	****	****	****	****	****	****
	LW vs. S	ns	ns	ns	ns	ns	**	**	****	****	****	****	****	****
	LW vs. SW	**	ns	ns	ns	ns	*	***	***	****	****	****	****	****
	S vs. SW	*	ns	ns	ns	ns	ns	ns	ns	ns	ns	ns	ns	ns
ACM	L vs. LW	ns	ns	ns	ns	ns	ns	ns	ns	ns	ns	ns	ns	ns
	L vs. S	*	ns	**	ns	ns	***	****	****	****	****	****	****	****
	L vs. SW	ns	ns	ns	ns	ns	***	****	****	****	****	****	****	****
	LW vs. S	**	ns	*	ns	ns	ns	****	****	****	****	****	****	****
	LW vs. SW	ns	ns	ns	ns	ns	ns	****	****	****	****	****	****	****
	S vs. SW	***	ns	ns	ns	ns	ns	ns	ns	ns	ns	ns	ns	ns

Note: *, **, ***, and **** represent statistical significance at *p* ≤ 0.05, 0.01, 0.001, and 0.0001, respectively. ns represents statistical non-significance: *p* > 0.05.

## Data Availability

Data are contained within the article.
